# Socioeconomic factors do not predict sleep apnea in a population sample from Mecklenburg-Western Pomerania, Germany

**DOI:** 10.1007/s11325-022-02614-1

**Published:** 2022-04-29

**Authors:** Markus Krüger, Anne Obst, Olaf Bernhardt, Ralf Ewert, Thomas Penzel, Beate Stubbe, Ingo Fietze, Tatyana Ivanovska, Reiner Biffar, Amro Daboul

**Affiliations:** 1 Zentrum Für Zahn-, Mund und Kieferheilkunde, Universitätsmedizin Greifswald, Greifswald, Germany; 2 Klinik Und Poliklinik Für Innere Medizin B, Universitätsmedizin Greifswald, Greifswald, Germany; 3 Department of Restorative Dentistry, Periodontology, Endodontology, Preventive Dentistry, and Pediatric Dentistry, University Medicine Greifswald, Greifswald, Germany; 4 Interdisziplinäres Schlafmedizinisches Zentrum, Charité – Universitätsmedizin Berlin, Berlin, Germany; 5 The Federal State Autonomous Educational Institution of Higher Education, I.M. Sechenov First Moscow State Medical University of the Ministry of Health of the Russian Federation, Moscow, Russia; 6 Fakultät Für Elektrotechnik, Medien Und Informatik, Ostbayerische Technische Hochschule Amberg-Weiden, Amberg, Germany

**Keywords:** Sleep apnea, Socioeconomic status, Apnea–hypopnea index, AHI, OSAS, Ordinal regression, Study of Health in Pomerania (SHIP)

## Abstract

**Purpose:**

Socioeconomic factors are known to modulate health. Concerning sleep apnea, influences of income, education, work, and living in a partnership are established. However, results differ between national and ethnic groups. Results also differ between various clinical studies and population-based approaches. The goal of our study was to determine if such factors can be verified in the population of Pomerania, Germany.

**Methods:**

A subgroup from the participants of the population-based Study of Health in Pomerania volunteered for an overnight polysomnography. Their data were subjected to an ordinal regressions analysis with age, sex, body mass index (BMI), income, education, work, and life partner as predictors for the apnea–hypopnea index.

**Results:**

Among the subgroup (N = 1209) from the population-based study (N = 4420), significant effects were found for age, sex, and BMI. There were no significant effects for any of the socioeconomic factors.

**Conclusion:**

Significant effects for well-established factors as age, sex, and BMI show that our study design has sufficient power to verify meaningful associations with sleep apnea. The lack of significant effects for the socioeconomic factors suggests their clinical irrelevance in the tested population.

## Introduction

Obstructive sleep apnea syndrome (OSAS) is a sleep disorder characterized by recurrent events of partial or complete collapse of the upper airways, leading to frequent episodes of apnea and hypopnea. Its signs and symptoms include snoring, sleep interruption, and excessive daytime sleepiness, which can lead to significant impairment in the quality of life of affected subjects. The disorder received increasing attention in the past decade, largely because of the growing research linking OSAS to cardiovascular disease [1] and to a decline in neurocognitive [2] and behavioral functions [3]. Population-based studies from different countries have also reported higher prevalence in recent years, which can be attributed in part to using more sensitive recording techniques and changes in scoring definitions [4, 5]. According to the current International Classification of Sleep Disorders (ICSD-3) [6], the diagnostic criteria for OSAS in adults should include either (1) five or more obstructive respiratory events (obstructive and mixed apneas, hypopneas, or respiratory effort-related arousals) per hour of sleep during an overnight polysomnography (PSG) setting or per hour of monitoring time using a home sleep apnea test (HSAT) with symptoms (daytime sleepiness, fatigue, insomnia, snoring, subjective nocturnal respiratory disturbance) and associated comorbidities or (2) fifteen or more predominantly obstructive respiratory events per hour of sleep during PSG or per hour of monitoring time during an HSAT. In most of those studies, OSAS severity has been indicated by the frequency of apnea and hypopnea events per hour of sleep (apnea–hypopnea index: AHI) as determined by overnight polysomnography.

Although the pathophysiology of the disorder is not completely understood and a multifactorial origin involving craniofacial and oropharyngeal anatomical abnormalities is suggested [7], there is evidence that increasing age, male sex, and obesity are risk factors for OSAS (see Punjabi, for an overview [8]). The association of age, sex, and BMI with AHI has been demonstrated previously for the sample of the current study [9]. Furthermore, a longitudinal study by Peppard et al. showed that weight reduction reduces symptoms of sleep apnea [10].

Concerning sex differences, previous studies showed that the severity of OSAS increases with age in both men and women, but men consistently have more severe symptoms and a higher AHI than women of the same age [11]. Furthermore, while earlier studies reported that OSAS is 8 to 10 times more prevalent in men [12, 13], recent prevalence reports showed OSAS in men to be 2 to 3 times more frequent than in women [4, 9].

### Income

A higher income connected with fewer symptoms of sleep apnea has been found in different national samples (e.g., Denmark [14]; Israel [15]; USA [16]), while in other samples, the connection was unclear (e.g., Brazil [17]; Greece [18]) or not detectable at all (e.g., France [19]).

It is unlikely that income influences sleep apnea directly. One commonly mentioned mediator might be BMI: At least in the industrialized world, access to food is universal, but people with lower income might only have access to lower quality food, leading to overnutrition and obesity, which in turn can lead to a higher incidence of sleep apnea in the economically disadvantaged. Socioeconomic status may also affect access to general medical services and to a calm and quiet living and sleeping environment.

However, the direction of effect may also point the other way. Suffering from sleep apnea might diminish an individual’s income-earning opportunities. This notion is in line with research findings that children afflicted by sleep apnea had a smaller estimated income in young adulthood and were more likely to depend on social support [14].

### Education/work

Occupational activity and working hours may be considered as contributing risk factors to OSAS. While some studies indicated that long working hours do not directly affect OSAS development, other studies showed OSAS risk from the mismatch between long working hours and low pay.

Subsequent studies have reexamined occupational factors across different job cohorts and demonstrated that workers exposed to high levels of job stress were more likely to have obstructive sleep apnea [20, 21]. From another perspective, increasing physical activity in sedentary jobs and reducing the time spent sitting during the day were associated with a decrease in AHI in OSAS patients, with more pronounced improvement in non-obese subjects.

One study found that occupational social class was associated with OSAS while education level was not, which reflects structural factors and exposure to further job-related psychosocial factors that are not associated with the level of education [22]. Nonetheless, more recent studies suggest that educational attainment in itself may be related to sleep quality and health in general, because education can enhance economic conditions, psychosocial resources, and lead to better health behaviors that can be linked to improved sleep quality [23].

### Life partner

In two studies, a higher incidence of sleep apnea in persons being married or cohabiting with a partner has been reported [19, 22]. It is speculated that dietary habits or psychosomatic factors are responsible. Another explanation might be that having sleep apnea is detrimental to having a life partner in the first place [24]. This in turn might lead to an underreporting of sleep apnea in singles, as there is no partner to observe snoring or episodes of apnea and hypopnea.

### Aims and scope

As the interdependencies between socioeconomic factors and sleep apnea are inconsistent across different clinical and non-clinical as well as national and ethnic samples, the goal of our present study was to test whether such factors could be verified in a population-based sample from Pomerania, Germany.

## Methods

### Sample

A subsample of the Study for Health in Pomerania (SHIP) was used in this study [25]. SHIP is a population-based study to monitor health parameters and their change over time in a longitudinal format with multiple cohorts. For the present study, the cohort SHIP-Trend was chosen [26], because for some of the participants an overnight polysomnography (PSG) in the local sleep laboratory was available. Further details on this specific SHIP subsample can be found elsewhere [9, 27]. The sample for SHIP-Trend was taken in the years 2008 to 2012. The 10,000 participants were randomly selected (stratified for age and sex) from official records, though only 8826 were reachable to be contacted, and of these 4420 participated in the basic study. A PSG was offered to all participants and 1264 accepted it. Complete AHI data were obtained for 1209 participants.

### Overnight polysomnography

An attended laboratory-based overnight polysomnography (PSG) was performed according to the manual of the American Academy of Sleep Medicine (AASM) [6] using ALICE 5 PSG devices (Philips Respironics, Eindhoven). Recordings included six electroencephalogram (EEG) channels, two electrooculogram (EOG) channels, two electromyogram (EMG) channels at chin and tibialis muscles, one electrocardiogram (ECG), a respiratory inductive plethysmography, nasal pressure sensor, pulse oximetry, a microphone to detect snoring, and a body position sensor.

Up to four subjects were recorded parallel per night, with 8 h of bedtime being requested (5 h of bedtime being the minimal requirement).

Sleep and breathing events were visually scored according to the 2007 AASM criteria: an apnea event was scored when there was a drop in peak signal excursion by ≥ 90% for at least 10 s, and with at least 90% of the event duration showing such amplitude reduction. A hypopnea event was scored if there was either flow drop of ≥ 30% for at least 10 s with ≥ 4% oxygen desaturation, or flow drop of ≥ 50% with ≥ 3% oxygen desaturation, or an arousal.

The apnea–hypopnea index (AHI) was used to define OSAS severity: AHI < 5 = normal, 5 ≤ AHI < 15 = mild, 15 ≤ AHI < 30 = moderate, and AHI ≥ 30 = severe [9, 27].

### Predictors

#### Equivalized income

Participants were asked in an interview whether their net monthly household income was below 500€, between 500€ and 900€, between 900€ and 1300€, between 1300€ and 1800€, between 1800€ and 2300€, between 2300€ and 2800€, between 2800€ and 3300€, between 3300€ and 3800€, or above 3800€. Arithmetic means for all ranges were computed. From the lower margin, 1/3 of the value was subtracted and to the higher margin 1/3 was added [28]. Values were then divided by the square root of the household size [29]. This is the standard procedure for the SHIP-Trend study analysis.

#### Estimated years of education

Years of education are estimated by combining participants’ answers to the questions about their highest qualification upon school graduation and their highest vocational qualification [28]. This estimate is specific for the German education system, where most qualifications are highly regulated.

#### Full-time work

Participants were asked about their employment status. If employed, they were asked the number of hours they usually worked per week. Working 37.5 h or more per week in a paid position or being self-employed with a similar work time counted as full-time work.

#### Life partner

Participants were asked whether they lived with a partner.

### Data analysis

Data analysis and visualization were conducted using R Statistics [30] and the packages haven [31], dyplr [32], tidyr [33], MASS [34], rstatix [35], and ggplot2 [36].

## Results

### Descriptive data

Of the 1209 participants, 559 (46%) were females, 542 (45%) were employed full-time, and 951 (79%) were living with a life partner (for further information on the relevant variables, see Table 1; a table with basic sleep parameters can be found in Appendix 1).

**Table 1:** Mean values, standard deviation (SD), and range of the continuous variables considered in the present study

Variable (units)	Mean	SD	Range
Age (years)	53	14	20–81
BMI (kg/m^2^)	28.4	4.9	18.4–52.9
Income (€/month)	1416.57	741.36	166.67–5066.67
Education (years)	13	2	0–17

(Note that income and education are pseudo-continuous).

**Fig. 1:**
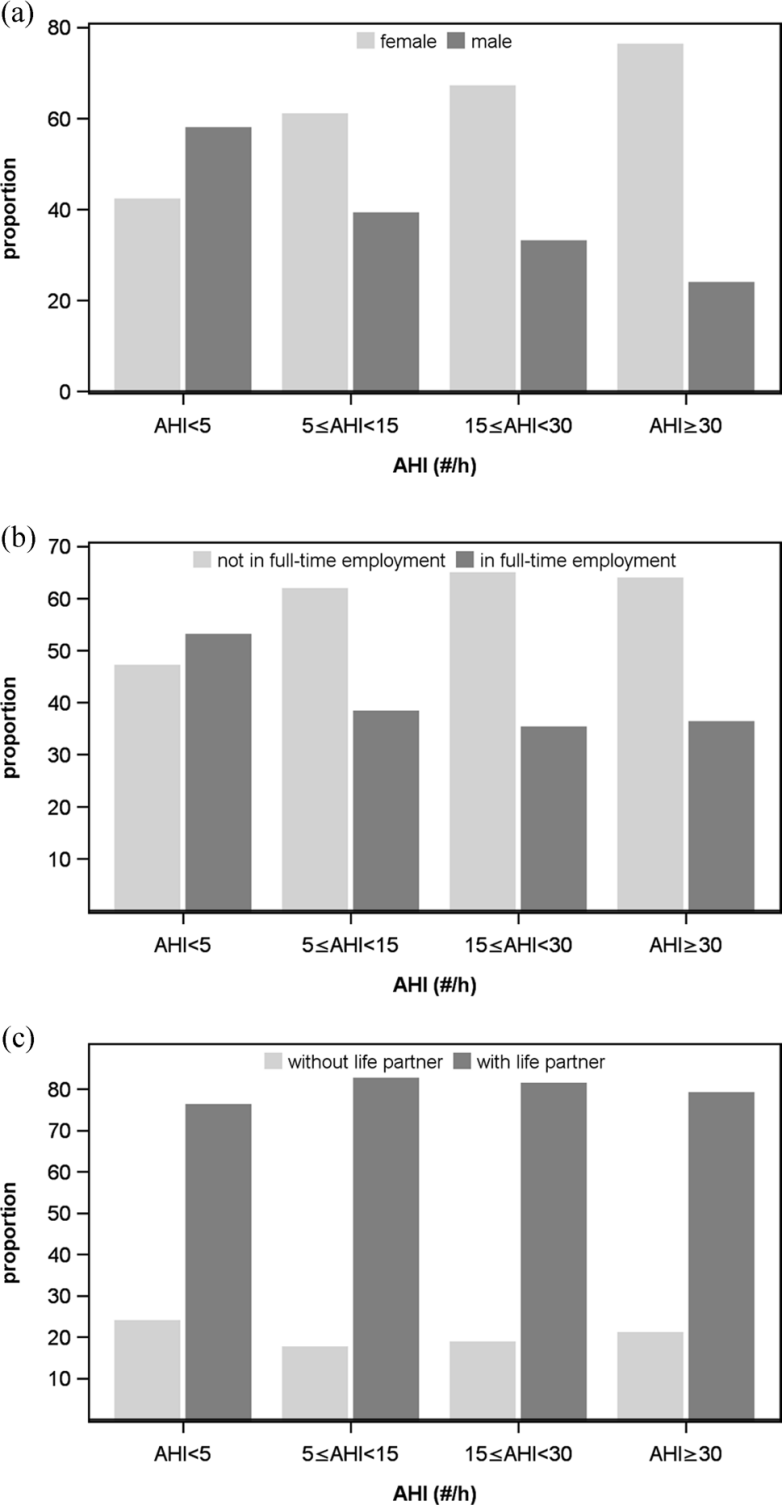
Proportions in percent of all the dichotomous variables **a** sex, **b** work, and **c** life partner for the categorized AHI (AHI < 5, normal; 5 ≤ AHI < 15, mild; 15 ≤ AHI < 30, moderate; AHI ≥ 30, severe)

**Fig. 2:**
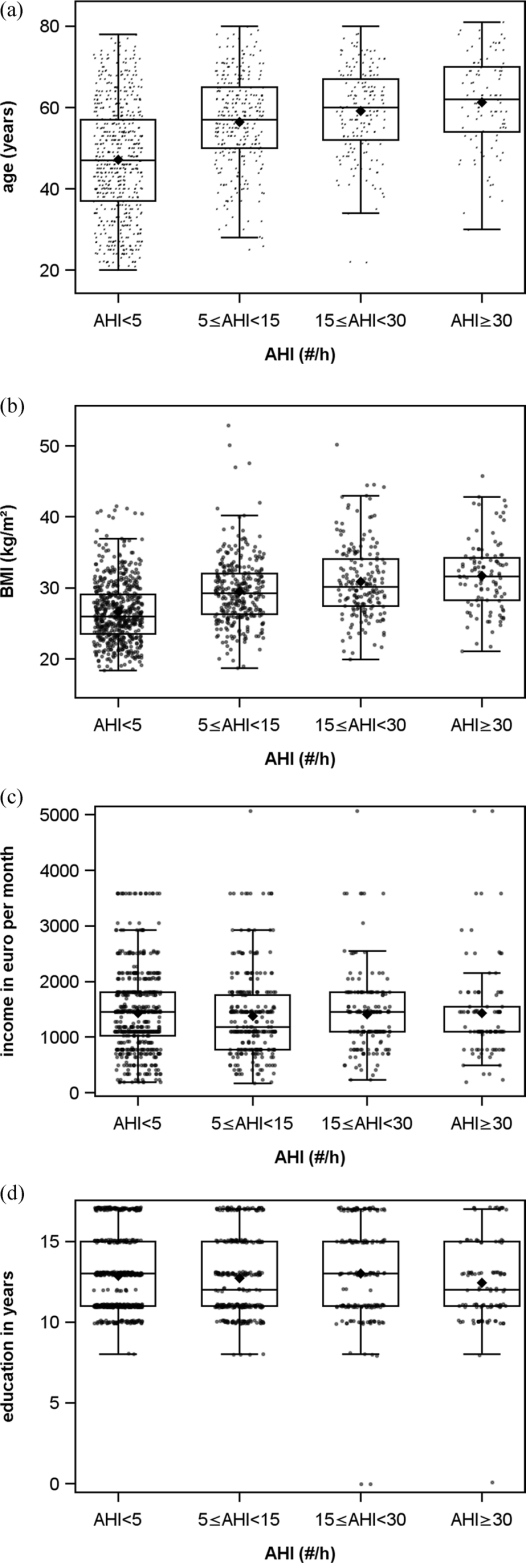
Boxplots with scattered data points of continuous variables **a** age in years, **b** BMI, **c** income in euro per month, and **d** education in years for the categorized AHI (AHI < 5, normal; 5 ≤ AHI < 15, mild; 15 ≤ AHI < 30, moderate; AHI ≥ 30, severe)

A visual inspection of the distributions for the different sleep apnea severity grades according to the AHI revealed the following: For dichotomous variables, it seems that the proportion of males to females increases with the grade of severity (Fig. 1a). The number of persons with full-time employment seems to diminish with the grade of severity; however, a clear-cut division is seen only between the categories “normal” and “mild” (Fig. 1b). There is no clear trend concerning the presence of a life partner (Fig. 1c). For continuous variables, there seems to be a higher median for every severity grade for age (Fig. 2a) and BMI (Fig. 2b), whereas there is no obvious trend discernable for income (Fig. 2c) or education (Fig. 2d).

### Ordinal regression analysis

An ordinal logistic regression with multiple dichotomous predictors (sex [female, male], work [not in full-time employment, in full-time employment], and life partner [no, yes]) and multiple continuous predictors (age [in years], BMI, income [in euro], and education [in years]) on AHI severity (AHI < 5, normal; 5 ≤ AHI < 15, mild; 15 ≤ AHI < 30, moderate; AHI ≥ 30, severe) was computed.

For the predictors, age, BMI, and sex significant effects were found, all *p* < 0.001. Each year of age increased the chance of a higher grade of severity by 7%, each unit in the BMI increased the chance by 15%, and being male by 253% when all other factors were held constant. (Note that the odd ratios in ordinal regressions are indicating the chance of being in the next higher category of the categorical target variable. This is in contrast to the logistic regression, where the target variable is dichotomous.) No significant results were found for the other predictors, all *p* > 0.10. An overview of the results can be found in Table 2.

**Table 2:** Odds, *p* values, and estimated effect sizes for all predictors

Predictor	Odds	*p*	η_p_^2^
Sex	3.53	< 0.001	0.06
Age	1.07	< 0.001	0.13
BMI	1.15	< 0.001	0.09
Work	1.03	0.84	0.0002
Life partner	0.86	0.34	0.004
Income	1	0.71	0.0002
Education	0.97	0.2	0.0008

### Effect sizes

To estimate the effect sizes, an ANOVA with the same variables as the ordinal regression was computed, but the dependent variable AHI was introduced as a continuous variable [37, 38]. Therefore the effect sizes may have been overestimated in relation to the regression analysis. Only the partial η^2^ was regarded. Effect sizes for the non-significant predictors of the ordinal regression were at least one exponent smaller than those of the significant ones. All results can be seen in Table 2.

### Bias

As submitting to a PSG was not required to participate in SHIP-Trend, it is possible that people with sleeping problems were more likely to consent to this additional examination. When asked informally, non-compliant participants reported that they were unwilling to undergo PSG because of time constraints. Fietze et al. [8] analyzed the current data set for differences between participants that took part in the PSG and those that declined. They found that the persons in the PSG-group were more likely males, older, had a higher BMI, a larger waist-to-hip ratio, consumed more alcohol, were more likely to smoke, more likely to be employed, had a higher education, slept more hours at night, had more trouble to fall asleep, and reported more snoring (see Table 3). However, when comparing inverse probability weighted data to not weighted data, no meaningful differences in the prevalence of OSAS were discernable. (Group comparisons concerning the predictors can be found in Appendices 2 and 3.)

**Table 3:** Contingency table for being aware of snoring and volunteering for the PSG. The proportion of participants undergoing the PSG did differ with their awareness of their own snoring, χ^2^(1, *N* = 4420) = 68.6, *p* < 0.001, indicating that persons aware of their own snoring chose the PSG proportionally more often. Overall, participants being aware that they were snoring on a regular basis chose the PSG more often (73%) than the others (64%)

		Snoring		
		NO	YES	
PSG	NO	2675	536	3211
	YES	872	337	1209
		3547	873	4420

## Discussion

While there was no significant effect found for the socioeconomic factors, the well-known predictors, sex, age, and BMI, showed highly significant associations with small to medium effects. These significant effects indicated that our model was sensitive for detecting meaningful factors. The lack of significant effects for socioeconomic factors does not demonstrate that socioeconomic factors play no role in sleep apnea, but strongly suggests there is no clinical relevance of them in our sample.

Our sample confirmed the higher prevalence of OSAS in men compared to women that has been previously reported in global studies [39]. On the one hand, some of the factors that might clarify the higher male risk could be attributed to the differences in upper airway anatomy, body fat distribution, and hormonal status [40, 41]. On the other hand, some studies have suggested that hormonal changes in women experiencing menopause may be responsible for an increase in AHI, and that menopause may even represent a better determinant than age for prevalence estimates [42, 43]. Furthermore, recent studies [44, 45] reported a significant increase in the incidence of OSAS in postmenopausal women, by which some studies postulated that weight gain that occurs after menopause, and anatomical and/or functional upper airway changes may account for the observed differences in OSAS prevalence and severity [46, 47].

Taking a look at the two studies in which a negative connection between sleep apnea and income was found, differences in conceptualization are apparent. The Danish sample of Jennum et al. [14] consisted of persons who showed symptoms of sleep apnea at a very young age. This design is highly selective, as sleep apnea becomes more prevalent in older age. In the study by Gilat et al. [15] income was operationalized in high and low, with the threshold being whether participants were paying social security tax. This might indicate an influence of deep poverty not represented in our German sample. In the French sample examined by Fuhrmann et al. [19], no influence of income was detectable. The strong health and social security system of France may be more similar to the German one. One might speculate that effects of income in regard to sleep apnea are leveled by preventing extreme poverty.

It is entirely possible that persons without life partners are not aware of their own symptoms of sleep apnea [24] and therefore can neither report it nor seek treatment.

Both studies that reported an effect of living with a partner relied on self-reporting [19] or had a sample of patients being referred to a polysomnography because of sleep-disordered breathing [22]. Such a bias should not appear in our population-based sample (but see “Strengths and limitations”). However, we, too, found that people being aware of their snoring are more likely to accept an offered PSG.

### Strengths and limitations

The strengths of the study include the large sample size, the consistent and standardized collection of exposure and outcome variables across the population-based SHIP cohort, and the use of validated and objective methods that are considered the current gold standard in sleep evaluation, including the polysomnography in an overnight supervised setting.

There are limitations that are inherent to the cohort and the design of this study. First, the cross-sectional design of this study does not allow any conclusions regarding causality. Longitudinal studies, although challenging, would provide superior evidence and might establish causality between baseline and follow-ups examinations.

Second, while adequate for our purpose, the ordinal regression analysis cannot determine local effects. Therefore, it is entirely possible that existing local effects concerning socioeconomic factors might not have been detected.

Third, the socioeconomic context of the population cohort we examined might have induced some selection bias and limited the generalizability of our results to the broader German population. That is because persisting socioeconomic disparities still exist between some of the areas included in our cohort and the rest of Germany [48].

Generally, a self-selection bias is to be expected in a population-based study. This is especially true for the subgroup that decided to partake in the additional PSG. Our own analysis and that by Fietze et al. [9] suggest that people aware of their own sleeping problems were more likely to consent to an overnight PSG.

## Conclusions and perspectives

In our population-based sample from Pomerania, Germany, effects of socioeconomic factors connected to sleep apnea were not verifiable. This finding does not invalidate findings of the other studies described, but highlights the need for even larger population-based studies, that ideally encompass multiple international regions measuring the same constructs with the same measurement tools.

## Appendix 1

**Table **

**Table 4:** Sleep parameters: *TIM* time in bed; *TST* total sleep time; *SE* sleep efficiency, percentage of total sleep time to total time in bed; *N1 (%)* percentage of sleep stage N1 related to total sleep time; *N2 (%)* percentage of sleep stage N2 related to total sleep time; *N3 (%)* percentage of sleep stage N3 related to total sleep time; *REM* *(%)* percentage of sleep stage REM related to total sleep time

	N	Minimum	Maximum	Median (25^th^; 75^th^)	Mean (± SD)
TIB (min)	1209	300	601	462 (431; 490)	460 (± 45)
TST (min)	1209	73	525	380 (335; 419)	374 (± 66)
SE (%)	1209	15.0	98.9	84.1 (75.1; 90.6)	81.4 (± 12.3)
N1 (%)	1209	3.9	90.8	24.3 (17.1; 35.8)	27.8 (± 14.4)
N2 (%)	1209	0.0	72.8	40.4 (32.1; 48.0)	39.9 (± 11.6)
N3 (%)	1209	0.0	53.6	18.2 (12.6; 24.0)	18.5 (± 8.7)

**4**

## Appendix 2

**Table **

**Table 5:** Mean values and standard deviation (SD) of the continuous variables for participants consenting to the PSG in contrast to those who did not. Differences are illustrated by the effect size Cohen’s *d*

Variable (units)	PSG (*N* = 1209) Mean (SD)	no PSG (N = 3,211) Mean (SD)	*d*
Age (years)	53 (14)	52 (16)	0.07
BMI (kg/m^2^)	28.4 (4.9)	28.0 (5.3)	0.008
Income (€/month)	1416.57 (741.36)	1340.78 (685.92)	0.11
Education (years)	13 (2)	12 (3)	0.39

**5**

## Appendix 3

**Table **

**Table 6:** Percent of participants in each category consenting to the PSG in contrast to those who did not

Variable	PSG (*N* = 1209)	No PSG (*N* = 3211)
Male	54%	46%
Work full time	45%	41%
Have life partner	79%	77%

**6**
